# Benders Decomposition Using Graph Modeling and Multi-Parametric Programming

**DOI:** 10.1021/acs.iecr.5c03189

**Published:** 2025-10-30

**Authors:** Parth Brahmbhatt, David L. Cole, Victor M. Zavala, Styliani Avraamidou

**Affiliations:** † Department of Chemical and Biological Engineering, 5228 University of Wisconsin-Madison, Madison, Wisconsin 53706, United States; ‡ Mathematics and Computer Science Division, Argonne National Laboratory, Lemont, Illinois 60439, United States

## Abstract

Benders decomposition is a widely used method for solving large and structured optimization problems, but its performance is affected by the repeated solution of subproblems. We propose a flexible and modular algorithmic framework for accelerating Benders decomposition. Specifically, we express the problem structure by using a graph-theoretic modeling abstraction in which nodes represent optimization subproblems and edges represent connectivity between subproblems. A key innovation of our approach is that we embed multiparametric programming (mp) surrogates for node subproblems, which maps the exact analytical map of the subproblem solution space. The use of mp surrogates allows us to replace subproblem solves with fast look-ups and function evaluations for primal and dual variables during the iterative Benders process. We formally show the equivalence between classical Benders cuts and those derived from the mp solution. We implement our framework in the open-source PlasmoBenders.jl software package. To demonstrate the capabilities of the proposed framework, we apply it to a two-stage stochastic programming problem, which aims to make optimal capacity expansion decisions under market uncertainty. We evaluate both single-cut and multicut variants of Benders decomposition and show that the use of mp surrogates achieves substantial speedups in subproblem solve time, while preserving the convergence guarantees of Benders decomposition. We highlight advantages in solution analysis and interpretability that is enabled by mp critical region tracking; specifically, we show that these reveal how decisions evolve geometrically across the Benders search. Our results aim to demonstrate that combining surrogate modeling with graph modeling offers a promising and extensible foundation for structure-exploiting decomposition. In addition, by decomposing the problem into more tractable subproblems, the proposed approach also aims to overcome scalability issues of mp. Finally, the use of mp surrogates provides a unifying and modular optimization framework that enables the representation of heterogeneous node subproblems as modeling objects with a homogeneous structure.

## Graphical abstract

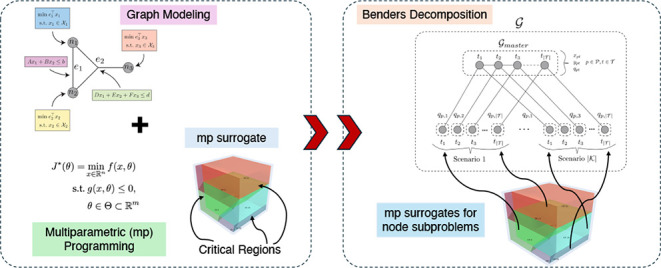

## Introduction

Many applications of industrial interest (e.g., energy and process planning/scheduling and supply chain design) often result in large and structured optimization problems. Classical monolithic formulations and solution strategies face computational challenges due to the size and complexity of such problems; as such, practitioners have relied on decomposition algorithms to obtain solutions that are tractable.^1–3^

Graph-theoretic modeling has shown to provide a modular framework that facilitates the expression and decomposition of structured optimization problems.^4,5^ Diverse graph abstractions have been proposed in the literature^6–16^ in this work we focus on on the OptiGraph abstraction introduced by Jalving and coworkers and implemented in the open-source package Plasmo.jl.^5^ Within the OptiGraph, optimization problems are represented as graphs composed of nodes containing optimization subproblems (holding local objectives, constraints, and variables) and edges (encoding linking constraints and variables that couple node subproblems).

A key observation that inspires our work is that graph modeling provides a flexible and modular foundation for the implementation of decomposition algorithms. Specifically, node subproblems can be isolated and replaced by surrogate models; in addition, tailored solution approaches for the node subproblems can be implemented all without requiring the model code to be rewritten. The graph approach also provides a framework that enables the implementation of tailored decomposition approaches.^4^

Benders decomposition^1,17^ is a classical and powerful decomposition approach that can be used to tackle problems that have an acyclic graph structure (e.g., trees encountered in stochastic programming). Moreover, we have recently shown that the approach can be applied to problems with general graph structures by conducting graph aggregation operations.^4^ A key aspect that limits the scalability of Benders decomposition is the repetitive solution of node subproblems. A promising approach to alleviate this is through the use of surrogate models for node subproblems.^18,19^ Surrogate models act as effective substitutes for expensive simulations or repetitive optimization solves. As a result, they can substantially reduce computational effort required in iterative algorithms such as Benders decomposition.^20^ In our context, surrogates can be used to reduce the computational burden of repeatedly solving graph subproblems. For instance, by learning the behavior of the subproblem solution map/policy, surrogates can quickly estimate optimal values or generate approximate cuts.

Recent work has extensively explored the use of machine learning (ML) models as surrogates to accelerate Benders decomposition across various domains,^14,21,22^ Many of these approaches use neural networks to approximate subproblem solutions and their duals to construct heuristic Benders cuts. Architectures range from standard feed-forward networks^23^ and Rectified Linear Unit (ReLU) networks^24^ to more specialized models like Graph Convolution Networks (GCNs), which generate cuts by exploiting the problem’s graph structure.^25^ Other strategies take different routes, such as using Input Convex Neural Networks (ICNNs) to directly replace the recourse function while maintaining convexity,^26^ or applying reinforcement learning (RL) to generate discrete actions for the master problem.^27^

Despite their success in reducing solution times, these ML-based surrogate approaches share limitations that motivate our work. The most significant drawback is the sacrifice of optimality guarantees; most methods are approximate or heuristic in nature, providing solutions quickly but forgoing the certificates of optimality that a converged Benders algorithm provides.^23,24,27^ While some studies preserve convergence guarantees by using ML classifiers to enhance cut management strategies rather than predict solutions^28–30^ they still require solving all repetitive scenario subproblems. Furthermore, many surrogate models require extensive offline training on large data sets^21,25,31^ and can be constrained by theoretical requirements, such as the convexity needed for ICNNs.^26^

Beyond computational performance, a key challenge in stochastic programming (SP) is that its solutions are often difficult for users to understand and trust, hindering practical adoption^32^ because of large number of scenarios. It is particularly difficult to explain *why* first stage decision is the best possible choice with uncertainty consideration.^32,33^ Classical Benders decomposition can exacerbate this by treating subproblems as black boxes, offering little insight into the interaction between master decisions and recourse actions. To address this challenge, researchers such as Rathi et al.^32^ have proposed systematic methods to enhance the explainability of SP solutions. These methods work by grouping scenarios together if they result in similar recourse decisions^34^ and by identifying the few key parameters that have the biggest impact on the solution. This process helps to clearly show the logic behind the optimal first-stage decision.

Multiparametric programming (mp) can be seen as a surrogate modeling approach that offers a interesting alternative to ML surrogates. By treating external variables and uncertain data as parameters, the entire family of node subproblems can be solved analytically once. The result is a collection of critical regions and associated affine solution functions that can be used to replace subproblem solves with fast critical region look-ups and vector-matrix multiplications to evaluate the affine functions at run time. Each critical region inherently represents a cluster of uncertain scenarios where the optimal strategy and active constraints are identical. This provides a direct, geometric answer to the explainability problem—instead of clustering scenarios after the fact, mp reveals the problem’s underlying decision structure analytically. While the mp approach has been leveraged in other contexts^35–38^ it has yet to be systematically integrated into Benders decomposition of graph-structured problems such as stochastic programs, largely because of scaling issues with an increasing number of scenarios.^39^ However, recent advances in fast mp solvers,^40^ combined with the availability of parallel computing resources and the use of decomposition schemes, enable the use of mp in large and structured problems. For instance, mp has been recently used for enabling the use of model predictive control in a network problems.^41^

In this work, we combine a graph modeling approach with multiparametric programming (mp) to accelerate Benders decomposition. By generating an exact analytical surrogate that completely maps the subproblem’s solution space, our method provides a rigorous algorithm with guaranteed convergence and a unique level of interpretability. We show that the classical Benders optimality cut and the affine value function produced by an mp-LP are formally identical when evaluated at the same active-set region. This equivalence guarantees that substituting mp surrogates for exact solves preserves the convergence of the Benders algorithm. While the one-time, offline solution of the mp-program is conceptually similar to the training phase of ML models, our approach offers distinct advantages over ML surrogates by providing a rigorous method with guaranteed convergence and a unique level of interpretability. Specifically, because the mp representation partitions the parameter space into polytopes, it offers an immediate, geometric interpretation of how successive Benders iterations migrate across critical regions,a level of transparency not attainable with ML based surrogate models. These benefits are achieved while still offering significant computational advantages relative to standard Benders. Moreover, the use of mp surrogates enables the standardization of node subproblems, thus streamlining modeling efforts and enabling plug-and-play of precomputed mp surrogates.

We implemented our framework using a combination of open-source software tools. We use Plasmo.jl to build the optimization model as a graph, PlasmoBenders.jl^4^ to manage the Benders decomposition steps, and a custom Python solver for mp^40,42^ that connects to the system. We focus our attention on tree structures that appear in stochastic programming problems. The resulting framework allows the modeler to declare stochastic mixed-integer linear programs as graphs, embed mp surrogates into graph nodes. Computational results in a capacity planning problem shows speed-ups—at times up to 2 orders of magnitude for subproblems—without sacrificing optimality guarantees. To the best of our knowledge, this is the first open-source implementation that integrates graph modeling and mp for Benders decomposition.

## Methods

### Graph Modeling

We represent structure optimization problems as a graph using the OptiGraph abstraction^5^ that is implemented in the Julia package Plasmo.jl. An OptiGraph is made up of a set of OptiNodes, N , and OptiEdges, E . An OptiNode contains an optimization problem (which its own objective, variables, data, and constraints); an OptiEdge contains constraints that link variables across OptiNodes. The OptiGraph abstraction, G , can be written as

1a $$\underset{\{x_{n}{\}}_{n\in N(G)}}{\min}f\left(\{x_{n}{\}}_{n\in N(G)}\right)\left(\mathrm{Objective}\right)$$

1b $$\mathrm{s.t.},x_{n}\in X_{n},n\in N\left(G\right),\left(\mathrm{Node Constraints}\right)$$

1c $$g_{e}\left(\{x_{n}{\}}_{n\in N(e)}\right)\geq 0,e\in E\left(G\right).\left(\mathrm{Link Constraints}\right)$$

where N(G) are the nodes on G , E(G) are the edges of G , N(e) are the nodes connecting edge *e*, ***x***_*n*_ are the decision variables on node *n*, and $X_{n}$ is the feasible set for the decision variables on node *n*. An example of the OptiGraph abstraction is shown in Figure 1.

**Figure 1:**
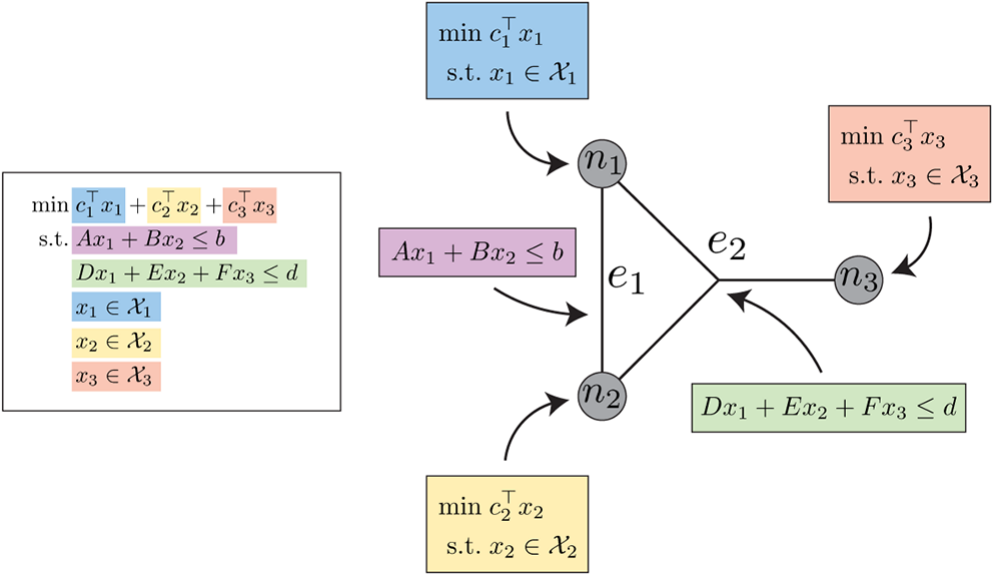
Example of OptiGraph abstraction, where the optimization problem on the left is partitioned into the nodes and edges of the graph on the right.

The OptiGraph abstraction is able to capture hierarchical relationships within problem structures and form nested subproblems.^4,5^ The ability to capture hierarchical structures makes the OptiGraph abstraction a flexible modeling tool to embed surrogate model for node subproblems. In principle, any subproblem node can contain or be replaced by a surrogate model, such as a mp program or a neural network. In this work, we will consider optimization problems where a node is replaced by a mp surrogate. The use of surrogate models to represent node subproblems provides an avenue to standardize the modeling of problems that contain heterogeneous components, as surrogates provide a unifying modeling abstraction for all components. This can help streamline modeling and gain insight into model and algorithmic behavior.

### Benders Decomposition

Benders Decomposition (BD)^1,17^ is a classical, iterative algorithm for solving structured linear programs (LP), mixed-integer linear programs (MILP), and to convex nonlinear problems. In classical BD, an optimization problem with a 2-level tree graph structure is decomposed into a master problem/node and one or more subproblems/subnodes, where the master problem contains a set of complicating variables that, once fixed, enable the parallel solution of subproblems. An iteration of BD includes solving the master problem, passing the primal variable solutions of the master problem to the subproblems, fixing those solutions in the subproblems, solving the subproblems to obtain primal and dual variables that are used for constructing cutting planes that are sent back to the master problem (see Algorithm 1). Each iteration of BD results in an upper bound obtained by the solution of the subproblems (a feasible solution) and in a lower bound obtained by solving the master problem (which contains a relaxation obtained using cutting planes).

In this work, we apply BD to structured LPs problems of the form:^4^

2a $$\min c_{m}^{T}x_{m}+\underset{w\in W}{\sum }c_{w}^{T}x_{w}$$

2b $$\mathrm{s.t.},x_{m}\in X_{m}$$

$$x_{w}\in X_{w},w\in W$$ 2c

2d $$C_{w}x_{m}+D_{w}x_{w}\leq q_{w},w\in W$$

Here, *x*_*m*_ are the master problem decision variables (complicating/coupling variables), *x*_*w*_ are the subproblem variables for subproblem *w*, *W* is the set of subproblems, $X_{w}$ is the linear feasible region of the subproblem *w*, and (*c*_*m*_, *c*_*w*_, *C*_*w*_, *D*_*w*_, *q*_*w*_) are problem data. The master problem has the form

$${\overset{¯}{\phi }}_{m}^{i}≔\min c_{m}^{T}x_{m}+\underset{w\in W}{\sum }\alpha_{w}$$ 3a

3b $$\mathrm{s.t.},x_{m}\in X_{m}$$

3c $$\alpha_{w}\geq \left\{\mathrm{cuts}\right\},w\in W$$

Here α_*w*_ are the subproblem cost variables that provide a lower bound to the optimal solution of the subproblems. Cuts are cutting planes that restrict the value that α can take. Subproblem *w* takes the form

4a $${\overset{―}{\phi }}_{w}^{i}\left({\mathrm{x̅}}_{m}^{i}\right)≔\min c_{w}^{T}x_{w}$$

4b $$\mathrm{s.t.},x_{w}\in X_{w}$$

4c $$C_{w}z_{w}+D_{w}x_{w}\leq q_{w}$$

4d $$z_{w}={\mathrm{x̅}}_{m}^{i}\left(\lambda_{w}^{i}\right)$$

Here, ${\mathrm{x̅}}_{m}^{i}$ are the solutions of the master problem at the *i*th iteration, *z*_*w*_ are variables that are fixed to the master problem solution, and $\lambda_{m}^{i}$ are dual variables corresponding to constraint (eq 14). Note that the constraint (eq 7) is enforced in the subproblem as (eq 13).

Because the master problem solution is enforced on the subproblem, the upper bound at iteration *i* is given by $UB^{i}=\underset{j\in \left\{1,...,i\right\}}{\min}c_{m}^{T}{\mathrm{x̅}}_{m}^{j}+\underset{w\in W}{\sum }c_{w}^{T}{\mathrm{x̅}}_{w}^{j}$ where ${\cdot ̅}^{j}$ corresponds to the optimal solution of that variable at iteration *j*. The lower bound at iteration *i* is given by $LB^{i}=c_{m}^{T}{\mathrm{x̅}}_{m}^{i}+\underset{w\in W}{\sum }{\mathrm{\alpha ̅}}_{w}^{i}$ . Finally, Benders cuts are added at each iteration and have the form, for iteration *i*, of $\alpha_{w}\geq {\mathrm{\phi ̅}}_{w}^{i}+\lambda_{w}^{iT}\left(x_{m}-{\mathrm{x̅}}_{m}^{i}\right)$ . This approach results in |*W*| cuts being added to the master problem at each iteration and is referred to as “multi-cuts”; an alternative approach is to use a single subproblem cost variable which is restricted by the sum of the right-hand side of the above cut for all subproblems, referred to as an aggregated or single cut.
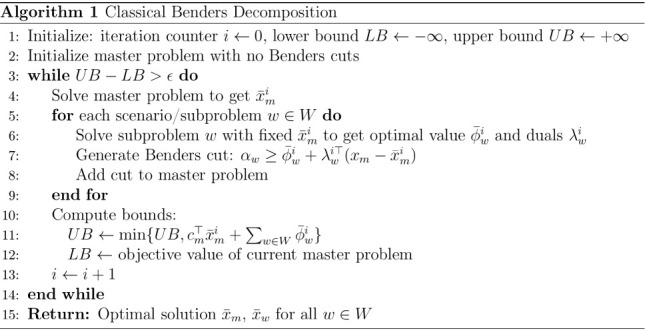


### Multiparametric Programming (Mp)

Multiparametric programming (mp)^43,44^ is an optimization technique that solves optimization problems as explicit functions of input parameters. It has gained significant attention in process systems engineering, particularly in model predictive control^37,44^ multilevel optimization^45,46^ and robust optimization.^38,47^ By parametrizing optimization problems, mp enables the derivation of optimal solution maps (for primal and dual variables) as functions of input parameters. Multiparametric programming aims to find the solution of the problem:

5a $$J^{*}\left(\theta \right)=\underset{x\in R^{n}}{\min}f\left(x,\theta \right)$$

5b s.t.⁣g(x,θ)≤0

5c $$\theta \in \Theta \subset R^{m}$$

where *x* is the vector of decision variables, θ is the vector of input parameters, *f*(*x*,θ) is the objective function, *g*(*x*,θ) represents the constraints, and Θ is a compact polytope representing the bounded uncertain parameters. The solution map of mp problems are characterized by critical regions (CR), which are subsets of the parameter space where a certain set of constraints is active. When the objective function is linear or quadratic and constraints are linear, these critical regions partition the feasible parameter space into polytopes, and within each region, the optimal solution is described by an affine function of the parameters. This structure allows for efficient implementation of optimal solution strategies, as the solution reduces to a simple function evaluation once the appropriate critical region is identified.

Here, we assume that the problem is an LP, but it is possible to apply mp to quadratic problems and MILP problems. The optimization formulation of a mp problem can be expressed as

6a $$z\left(\theta \right)=\underset{x}{\min}c^{T}x+\theta^{T}H^{T}x$$

6b s.t.⁣Ax≤b+Fθ

6c $$A_{\mathrm{eq}}x=b_{\mathrm{eq}}+F_{\mathrm{eq}}\theta$$

6d $$\theta \in \Theta ≔\left\{\theta \in R^{q}|A_{\theta }\theta \leq b_{\theta }\right\}$$

6e $$x\in R^{n}$$

Here, the objective function is linear in the decision variables *x* and is influenced by the parameters θ through the term $\theta^{T}H^{T}x$ . The constraints *Ax* ≤ *b* + *F*θ and *A*_eq_ *x* = *b*_eq_ + *F*_eq_θ show that the right-hand side of the inequalities and equalities are affine functions of the parameters θ. The final constraint, θ ∈ Θ, defines the space of feasible parameters as a polytope defined by the linear inequalities *A*_θ_θ ≤ *b*_θ_. This formulation seeks an optimal solution *x* for every possible value of θ within its defined bounds Θ.

The explicit (mp) solution to eq 18 can be expressed as

7 $$\begin{array}{ll} x^{*} & =A^{v}\theta +b^{v}\;if\;CR^{v}:E^{v}\theta \leq f^{v}\\ \forall v & =1,2,3,...,n_{CR} \end{array}$$

where x* is the solution of the problem, *A* and *b* represent the affine function defined for each critical region *v*, with *E* ^*v*^ and *f* ^*v*^ as the corresponding inequality matrices that define the critical region polytopes, and *n*_CR_ denotes the total number of critical regions.

### Mp Surrogates for Benders Subproblems

To demonstrate the utility of mp-based surrogates for Benders decomposition, we focus on two-stage stochastic programming problems (2-level tree graph structure). However, the proposed approach is general and can be applied to Benders decomposition of more general graphs. In a decomposition framework, the problem is split into a master problem and scenario-specific subproblems. For each scenario *k*, the subproblem (eq 11) is defined separately and must be solved repeatedly during each iteration of the Benders algorithm. When the subproblem contains scenario-dependent data such as stochastic objective coefficients and right-hand side values, these elements can be treated as parameters. This allows the subproblem to be reformulated as a multiparametric linear program (mp-LP), where the parametric dependence is explicitly modeled. It is worth noting that the same methodology is applicable when the subproblem is a multiparametric mixed-integer linear program (mp-MILP).

Consider a scenario-specific subproblem (eq 11) can be redefined as

8a $${\overset{―}{\phi }}_{w,k}^{i}\left({\mathrm{x̅}}_{m}^{i}\right)≔\min{\left[\begin{array}{l} \left[c_{f\;}0\right]\\ \left[0\;c_{w,k}\right] \end{array}\right]}^{T}x_{w}$$

8b $$\mathrm{s.t.},A_{f}x_{w}\leq b_{f}$$

8c $$C_{f}z_{w}+D_{f}x_{w}\leq \left[\begin{array}{l} q_{f}\\ q_{w,k} \end{array}\right]$$

8d $$z_{w}={\mathrm{x̅}}_{m}^{i}\left(\lambda_{w}^{i}\right)$$

Here, *c*_*f*_ and *q*_*f*_ are the fixed (nonrandom) components, while *c*_*w,k*_ and *q*_*w,k*_ capture the scenario-dependent (random/uncertain) components. This formulation assumes fixed recourse, meaning the constraint matrices (*A*_*f*_, *C*_*f*_, *D*_*f*_) are deterministic. Extending the framework to handle scenario-dependent Left-Hand Side (LHS) parameters (e.g., *D*_*w*_) is significantly more challenging because it introduces nonconvex bilinear terms of the form “parameter” × “variable” (e.g., *D*_*w*_ *x*_*w*_). A standard technique to handle such terms is to discretize the uncertain parameter: its continuous range is represented by a finite set of discrete points, and binary variables are introduced to select which point is active. A Big-M reformulation is then used to linearize the product, effectively transforming the subproblem from an mp-LP into a mpMILP. While solving an mp-MILP is more computationally intensive, the overall mp-Benders framework remains applicable. The linking variables *z*_*w*_ are coupled to the master problem solution ${\mathrm{x̅}}_{m}^{i}$ through an equality constraint. This subproblem can be reformulated as a mp-LP where the parameter vector comprises the first stage variables and the uncertain data:

$${\overset{¯}{\phi }}_{w,k}^{i}\left({\mathrm{x̅}}_{m}^{i}\right)≔\min{[c_{f}\;0]}^{T}x_{w}+{[0\;c_{w,k}]}^{T}I^{T}x_{w}$$ 9a

9b $$\mathrm{s.t.},A_{f}x_{w}\leq b_{f}$$

9c $$D_{f}x_{w}+C_{f}z_{w}\leq q_{f}+Iq_{w,k}$$

9d $$z_{w}=I{\mathrm{x̅}}_{m}^{i}\left(\lambda_{w}^{i}\right)$$

Here *I* is the identity matrix. Let $x=\left[\begin{array}{l} x_{w}\\ z_{w} \end{array}\right]$ be the full decision variable vector, and define the parameter vector as

$$\theta =\left[\begin{array}{l} {\mathrm{x̅}}_{m}^{i}\\ c_{w,k}\\ q_{w,k} \end{array}\right]$$

Then, the problem in (eq 28) fits the standard mp-LP structure described in (eq 18), with the following definitions:

$$\begin{array}{l} c=\left[\begin{array}{l} {\left[0\;c_{f}\right]}^{T}\\ 0 \end{array}\right],H=\left[\begin{array}{lll} 0 & 0 & 0\\ I & 0 & 0 \end{array}\right],\\ A=\left[\begin{array}{ll} A_{f} & 0\\ D_{f} & C_{f} \end{array}\right],b=\left[\begin{array}{l} b_{f}\\ q_{f} \end{array}\right],\\ F=\left[\begin{array}{lll} 0 & 0 & 0\\ 0 & 0 & I \end{array}\right],A_{\mathrm{eq}}=\left[\begin{array}{ll} 0 & I \end{array}\right],\\ b_{\mathrm{eq}}=0,F_{\mathrm{eq}}=\left[\begin{array}{lll} I & 0 & 0 \end{array}\right]. \end{array}$$

It is important to note that all components used in defining these matrices such as **0** and *I* are themselves matrices of appropriate dimensions. Therefore, the overall matrices A, H, F, *A*_eq_, and *F*_eq_ are block matrices. It is important to emphasize that for the two-stage stochastic programs considered in this work, all scenario subproblems are structurally identical; they differ only in the realized values of the uncertain data, which are captured by the parameter vector θ. This homogeneity is a significant advantage, as it allows for a single mp-surrogate to be generated once in an offline step and then reused for every subproblem solve across all Benders iterations. For more general problems decomposable by Benders where subproblems might be structurally distinct (e.g., arising from different physical units or time periods with different models), a separate mp-surrogate would need to be computed for each unique subproblem structure, increasing the computational burden.

#### Equivalence Between Benders Cuts and Mp-Based Cuts

At a given Benders iteration *i*, after the master problem yields a solution ${\mathrm{x̅}}_{m}^{i}$ , the goal is to generate an optimality cut. The classical Benders cut is a linear approximation of the subproblem’s value function, formulated as

$$\alpha_{w}\geq {\mathrm{\phi ̅}}^{i}+\lambda_{w}^{iT}\left(x_{m}-{\mathrm{x̅}}_{m}^{i}\right)$$

To construct this cut, two key pieces of information are required from the subproblem: 1. The optimal objective value, ${\mathrm{\phi ̅}}^{i}$ . 2. The optimal dual variables, $\lambda_{w}^{i}$ , corresponding to the constraints involving the master variables.

Our objective is to demonstrate that the mp surrogate can provide both of these components without needing to solve a new LP, thus proving the cuts are identical. Obtaining the subproblem optimal value from the mp solution is straightforward. From the mp solution of the subproblem, the parametric objective function is given by

$$\phi \left(\theta \right)=c^{T}x+\theta^{T}H^{T}x$$

For the current set of parameters θ̅ (which includes ${\mathrm{x̅}}_{m}^{i}$ ), we first identify its corresponding critical region, *v*. The optimal value ${\mathrm{\phi ̅}}^{i}$ is then found by a simple and computationally fast evaluation of the precomputed affine function for that region:

10 $${\mathrm{\phi ̅}}^{i}=c^{T}\left(A^{v}\mathrm{\theta ̅}+b^{v}\right)+{\mathrm{\theta ̅}}^{T}H^{T}\left(A^{v}\mathrm{\theta ̅}+b^{v}\right)$$

A crucial property of mp-LPs is that the dual variables $\left(\lambda_{w}^{i}\right)$ , like the primal solution, is a piecewise affine function of the parameters θ. We can derive this function explicitly by analyzing the subproblem optimality conditions. By definition, within a single critical region, the set of active constraints at the optimal solution is constant. This allows us to formulate the subproblem as an equivalent equality-constrained LP for that specific region:

11a $$\underset{x}{\min}c^{T}x+\theta^{T}H^{T}x$$

11b $$\mathrm{s.t.},A_{AS}x=b_{AS}+F_{AS}\theta$$

here, *A*_*AS*_, *b*_*AS*_, and *F*_*AS*_ contain only the rows from the original problem matrices that correspond to the active constraints in region *v*. To find the duals, we examine the Karush–Kuhn–Tucker (KKT) conditions for this problem. The Lagrangian function is

12 $$L\left(x,\lambda ,\theta \right)={\left(H\theta +c\right)}^{T}x+\lambda^{T}\left(A_{AS}x-b_{AS}-F_{AS}\theta \right)$$

The first-order KKT condition for optimality (stationarity) requires the gradient of the Lagrangian with respect to *x* to be zero:

13 $$\nabla_{x}L\left(x,\lambda ,\theta \right)=H\theta +c+A_{AS}^{T}\lambda =0$$

From this stationarity condition, we can directly solve for the dual variables λ as an explicit function of the parameters θ:

$$\lambda \left(\theta \right)=-{\left(A_{AS}^{T}\right)}^{-1}\left(H\theta +c\right)$$

This equation shows that the dual variables are an affine function of the parameters, which we can write as λ­(θ) = Λ^*v*^ θ + δ^*v*^, where the matrix $\Lambda^{v}=-{\left(A_{AS}^{T}\right)}^{-1}H$ and the vector $\delta^{v}=-{\left(A_{AS}^{T}\right)}^{-1}c$ are constant for each critical region *v*.

With explicit affine functions for both the objective value and the dual variables, we can now construct the final mp based cut. By evaluating the function for λ­(θ) at the current parameter vector ${\mathrm{\theta ̅}}^{i}$ , we get the required duals $\lambda_{w}^{i}$ . Substituting this into the standard Benders cut formula gives the explicit mp-based affine cut:

14 $$\alpha_{w}\geq {\mathrm{\phi ̅}}^{i}+{\left({\Lambda^{v}\mathrm{\theta ̅}}^{i}+\delta^{v}\right)}^{T}\left(x_{m}-{\mathrm{x̅}}_{m}^{i}\right)$$

This derivation formally establishes that the mp-based cut is identical to the classical Benders cut. This equivalence is important, as it guarantees that our surrogate-based method preserves the theoretical convergence of Benders decomposition while replacing expensive LP solves with fast function evaluations.

### Software Framework

The algorithmic framework proposed was implemented in open source software available at https://github.com/Avraamidou-Research-Group-CESE/MP-Plasmo-Benders. The framework relies on Python (for the mp-surrogates) and Julia (for graph-based modeling and Benders decomposition). The integration of two different programming environments highlights the modularity that is enabled by using mp surrogates within a graph modeling environment. Below we outline the implementation of the framework.

We used the PPOPT toolbox^40^ in Python to generate the mp solution. The subproblem eq 28 can be formulated and solved as a mp problem after defining parameters. The solution of the resulting mp problem contains *A* ^*v*^ and *b* ^*v*^ that represent the affine function defined for each critical region *v*, with *E* ^*v*^ and *f* ^*v*^ as the corresponding inequality matrices that define the critical region as shown in eq 23. This list of matrices for each critical region, representing the entire map of optimal solutions of the subproblem eq 28 for any realization of the defined parameters, is saved and loaded to Julia.

After loading the mp model solution, we build the full optimization problem in Plasmo.jl.^5^ The master problem and each subproblem are placed on independent subgraphs with linking constraints between the master problem and each subproblem (Figure 2). Each subproblem is independent of the other subproblems such that each linking constraint only links the master problem subgraph and each subproblem subgraph. Once this structure has been created, Benders decomposition can be applied via the package PlasmoBenders.jl. This package takes the user-defined OptiGraph structure and requires the user to set the master subgraph. Once this subgraph is set, PlasmoBenders.jl detects the downstream subgraphs, fixes the complicating variables in the subproblem subgraphs, and adds the cost to go variable (s). In the case of Figure 2, the user can set subgraph $G_{0}$ as the master problem and PlasmoBenders.jl will recognize that subgraphs $G_{1}$ - $G_{N}$ are the subproblem subgraphs.

**Figure 2:**
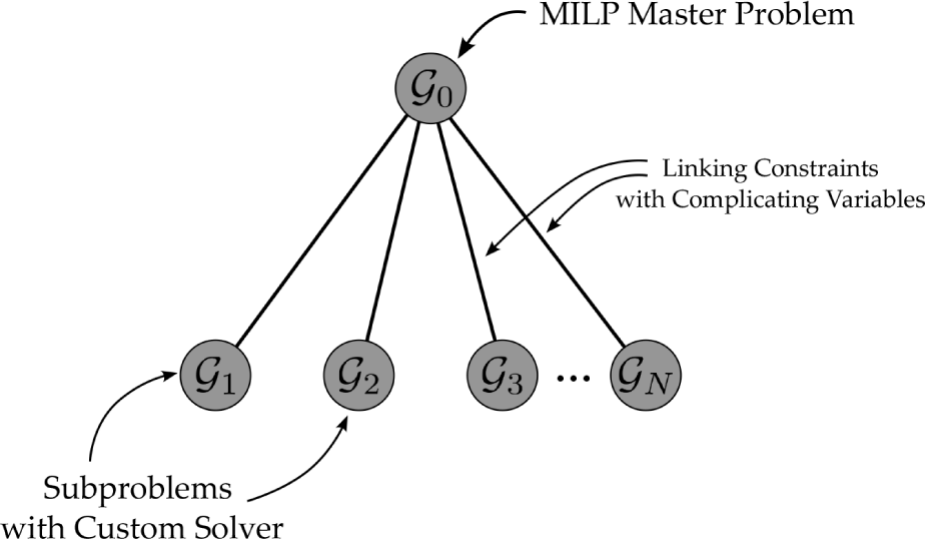
Graph representation of the problem solved with Benders. The master problem is stored on a subgraph, $G_{0}$ , and each subproblem is stored on independent subgraphs $G_{1}$ - $G_{N}$ . Edges contain the constraints with complicating variables.

To implement the mp surrogate models within the graph modeling framework, we define a custom solver in Julia such that no changes are required to the existing software of Plasmo.jl and PlasmoBenders.jl. This is a “custom solver” in the sense that we have defined our own methods in the Julia code that uses the mp data to solve the optimization problem and query primal-dual information, independent of typical LP solvers such as HiGHS or Gurobi. This custom solver interfaces with JuMP.jl’s MathOptInterface.jl (used in the backend of Plasmo.jl) to query primal-dual information from mp surrogate data that was determined from PPOPT toolbox. The user configures the solver as the optimizer for the subproblem corresponding to a specific subgraph and subsequently retrieves the mp solution data directly from the solver. Once optimize! is called on the subproblem, primal and dual information queried from the solver will be returned internally from the mp solution functions. By creating the custom solver, we allow for flexibly working with existing, registered packages and provide a framework for handling surrogate modeling approaches within the graph-based modeling framework.

The custom solver identifies the active critical region for a given parameter vector θ̅ by performing a linear scan. Specifically, it iterates through all precomputed regions and checks the corresponding inequalities *E* ^*v*^θ̅ ≤ *f* ^*v*^. While this naive search can become a bottleneck for problems with a very large number of critical regions, we accelerate the process using performance features in Julia such as the @fastmath macro. We avoided more complex data structures (e.g., binary search trees^48^ or linear machine^49^ due to their high offline computational cost to generate. This trade-off motivates the hierarchical surrogate approach discussed as future work.

The overall outline of the proposed framework is summarized in algorithm 2 below. 
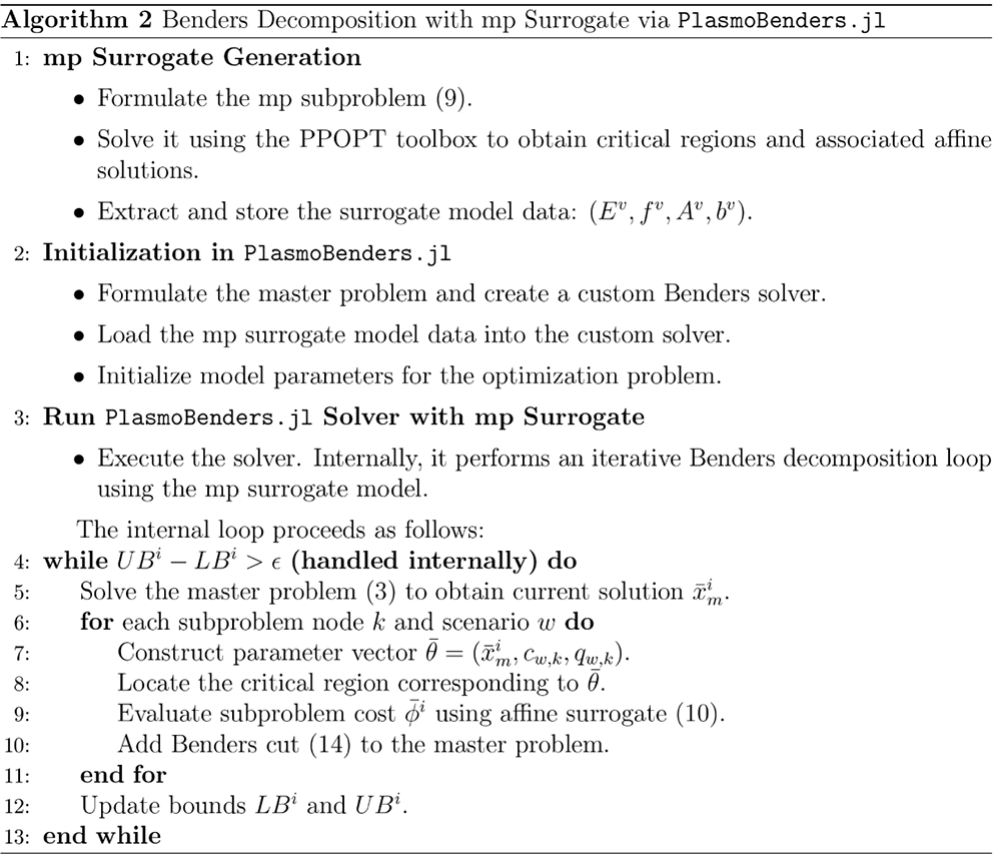
Replacing LP solves with simple affine evaluations makes the subproblem phase almost cost-free while preserving convergence guarantees, as the cuts are unchanged.

## Case Study: Capacity-Expansion Planning Under Uncertainty

To highlight how the proposed methodology and software framework can be applied, we present a case study of a capacity-expansion planning problem under uncertainty for a chemical process.^50^ The capacity of the chemical process units can be expanded at different points in time, while there is market uncertainty in the expected demands, availabilities, and prices of chemicals. The first-stage (master problem) variables represent capacity expansion decisions. The subproblem variables capture the purchase of raw materials, as well as the production and sale of intermediate and final products. While this case study is specific to a chemical process, the mathematical structure of this problem is common in many applications and domains, including power infrastructure planning^51^ and energy systems planning.^52^

### Problem Description

In its most basic form, the long-term capacity expansion and production planning problem with fixed-charge expansion costs can be stated as follows. Given an extended network of continuous production processes, *p*, and chemicals, *j*, the primary goal is to determine the optimum selection of processes and how their capacities should be expanded over a future horizon consisting of a set of time periods, *t*. Over this planning horizon, the demands, availabilities and prices of the chemicals are forecast as stochastic quantities, while the process capital investment, operating costs and expansion bounds, are deterministically known. Information is also given on how each process converts the raw materials into products in terms of conversion factors.

Because of the presence of uncertainty in the market conditions, the expected demands, availabilities and prices of the chemicals are predicted according to a set of scenarios, k∈K . To manage this uncertainty, the decisions are grouped into a couple of stages. In the absence of knowing the uncertainty, the capacity expansion decisions have to be made in the *first stage*, irrespective of which scenario occurs. However, the production rates of the processes and the amounts of chemicals purchased and sold can be manipulated in the *second stage* to best satisfy the market conditions revealed under each scenario.

The objective is to identify the optimal capacity plan to implement that maximizes the expected profit while still ensuring feasible plant operations over all the uncertain future realizations. The decision whether an expansion should take place or not is modeled through the use of a binary variable. The model is, therefore, formulated as a MILP problem (CEP) with the notation presented in eq 38.

15a $$\underset{x_{pt},y_{pt},q_{pt}}{\min}\underset{p\in P}{\sum }\underset{t\in T}{\sum }\left[\alpha_{pt}x_{pt}+\beta_{pt}y_{pt}\right]+E_{\xi }\left[V\left(q,\xi \right)\right]$$

15b $$\begin{array}{ll} \mathrm{s.t.},E_{pt}^{L}y_{pt}\leq x_{pt}\leq E_{pt}^{U}y_{pt} & \forall p\in P,t\in T \end{array}$$

15c $$\begin{array}{ll} 0\leq q_{pt}\leq Q_{pt}^{U} & \forall p\in P,t\in T \end{array}$$

15d $$\begin{array}{ll} q_{pt}-q_{pt-1}-x_{pt}=0 & \forall p\in P,t\in T \end{array}$$

15e $$\begin{array}{ll} x_{pt}\geq 0 & \forall p\in P,t\in T \end{array}$$

15f $$\begin{array}{ll} y_{pt}\in \left\{0,1\right\} & \forall p\in P,t\in T \end{array}$$

Here, *x*_*pt*_ are capacity expansion decisions, *q*_*pt*_ are cumulative expansion decisions, and *y*_*pt*_ are binary decisions to expand or not at given time. The objective (eq 38) is the minimization of the total cost, composed of the first-stage investment costs and the expected recourse cost from the second stage, $E_{\xi }\left[V\left(q,\xi \right)\right]$ . The investment costs include a variable cost component, α_*pt*_ *x*_*pt*_, which is proportional to the size of the expansion, and a fixed-charge cost, β_*pt*_ *x*_*pt*_, which represents a capital cost incurred only if the decision to expand is made. The model is formulated as a MILP precisely to handle this fixed-charge cost. Constrains (eq 39) restricts within physical limits the capacity expansion of processes. In the same constrains, the binary variables, *y*_*pt*_, denote the time *t* that capacity expansion takes place. Constrains (eq 40) bound the total available capacity to the maximum amount allowed $\left(Q_{pt}^{U}\right)$ and enforce nonnegative capacities. Equality constrains (eq 41) add new expansions to the already available capacities from pervious time periods. Finally, constraints (eq 42) enforce non-negativity for capacity variables, and constraints (eq 43) define binary indicator constraints.

For a fixed first-stage plan *q*_*pt*_ and realization $\xi =\left(A_{jt}^{k},D_{jt}^{k},\gamma_{jt}^{k},\varphi_{jt}^{k}\right)$ , the recourse cost variable, *V*(*q*,ξ), is calculated based on the optimization problem (eq 44).

16a $$V\left(q,\xi \right)=\underset{\begin{array}{l} p_{pt},b_{jt},s_{jt} \end{array}}{\min}-\underset{t\in T}{\sum }\left[-\underset{p\in P}{\sum }\sigma_{pt}p_{pt}+\underset{j\in J}{\sum }\gamma_{jt}b_{jt}-\underset{j\in J}{\sum }\varphi_{jt}s_{jt}\right]$$

16b $$\begin{array}{ll} \mathrm{s.t.},p_{pt}-q_{pt}\leq 0 & \forall p,t \end{array}$$

16c $$\begin{array}{ll} s_{jt}\leq A_{jt},b_{jt}\leq D_{jt} & \forall j,t \end{array}$$

16d $$\begin{array}{ll} p_{pt}\geq 0,s_{jt},b_{jt}\geq 0 & \forall p,j,t \end{array}$$

16e $$\begin{array}{ll} \underset{p\in P}{\sum }\left(\mu_{jp}-\eta_{jp}\right)p_{pt}+b_{jt}-s_{jt}=0 & \forall j,t \end{array}$$

Once market and technological uncertainties are realized, the plant operates at recourse production levels $p_{pt}^{k}$ , with external purchases $b_{jt}^{k}$ and sales $s_{jt}^{k}$ of intermediates and products. These decisions are determined as the optimal solution of the subproblem (eq 44). The recourse function is defined by the objective (eq 44), which minimizes the negative of the profit. Constraints (eq 45) ensure that production does not exceed the available capacity. Constraint (eq 46) restricts the quantities of intermediates and final products that can be bought or sold. Finally, constraint (eq 48) enforces material balance by ensuring that the total amount produced and purchased equals the amount consumed or sold.

To illustrate the scale of the optimization problem, we provide the generalized expressions for the number of variables and constraints in the full monolithic formulation, where *T* is the total number of time periods, *P* is the number of processes, *J* is the number of chemicals, and *K* is the number of scenarios.

- **Number of continuous variables** = *T* × (2*P* + *K* × (2*J* + *P*))
- **Number of binary variables** = *T* × *P*
- **Number of inequality constraints** = *T* × (5*P* + *K* × (4*J* + 2*P*))
- **Number of equality constraints** = *T* × (*P* + *J* × *K*)

Each of the *K* Benders subproblems is significantly smaller. For a single time period and scenario, the subproblem has *T* × (*P*+2*J*) continuous variables, *T* × (2*P*+4*J*) inequality constraints, and *T* × *J* equality constraints.

### Benders Reformulation

To embed the stochastic capacity-expansion planning framework with master problem (eq 38) and recourse model (eq 44) into a Benders decomposition framework, a couple of variants are considered: a *multicut* scheme, which generates one cut for each scenario-period pair (k,t)∈K×T per iteration, and a *single-cut* scheme, which aggregates all cuts into a single inequality. Let π_*k*_ denote the probability of scenario *k*, and assume uniform probability $\pi_{k}=\frac{1}{\left|K\right|}$ for all k∈K . The formulation is designed to accommodate a relatively complete recourse structure for this problem. Below the problem formulation of the master and subproblems is presented.

Master problem at iteration *l* can be given by formulation eq 54. For BD, we introduce auxiliary variables α_*kt*_ (multicut) or a single variable α (single-cut) to approximate the recourse cost. The master problem in the multicut form is then given by

17a $$\underset{x,y,q,\alpha_{kt}}{\min}\underset{p\in P}{\sum }\underset{t\in T}{\sum }\left[\alpha_{pt}x_{pt}+\beta_{pt}y_{pt}\right]+\underset{k\in K}{\sum }\underset{t\in T}{\sum }\pi_{k}\alpha_{kt}$$

17b $$\begin{array}{ll} \mathrm{s.t.},E_{pt}^{L}y_{pt}\leq x_{pt}\leq E_{pt}^{U}y_{pt} & \forall p,t \end{array}$$

17c $$\begin{array}{ll} 0\leq q_{pt}\leq Q_{pt}^{U} & \forall p,t \end{array}$$

17d $$\begin{array}{ll} q_{pt}-q_{p,t-1}-x_{pt}=0 & \forall p,t \end{array}$$

17e $$\begin{array}{ll} y_{pt}\in \left\{0,1\right\},x_{pt}\geq 0 & \forall p,t \end{array}$$

17f $$\begin{array}{ll} \alpha_{kt}\geq {\mathrm{\phi ̅}}_{kt}^{l}+{\lambda_{kt}^{l}}^{T}\left(q_{pt}-{\mathrm{q̅}}_{pt}^{l}\right) & \forall k,t,l=1,...,i \end{array}$$

In the single-cut formulation, only the objective and the cut constraint are modified as follows:

Objective (replace (eq 49)):

$$\underset{x,y,q,\alpha }{\min}\underset{p\in P}{\sum }\underset{t\in T}{\sum }\left[\alpha_{pt}x_{pt}+\beta_{pt}y_{pt}\right]+\alpha$$

Cut constraint (replace (eq 54)):

$$\alpha \geq \underset{k\in K}{\sum }\underset{t\in T}{\sum }\pi_{k}\left[{\mathrm{\phi ̅}}_{kt}^{l}+{\lambda_{kt}^{l}}^{T}\left(q_{pt}-{\mathrm{q̅}}_{pt}^{l}\right)\right]\forall l=1,...,i$$

Here ${\mathrm{q̅}}_{pt}^{l}$ is master solution at iteration l , ${\mathrm{\phi ̅}}_{kt}^{l}$ is optimal value of the (*k*, *t*) subproblem at iteration l , and $\lambda_{kt}^{l}$ is dual multipliers from the subproblem at iteration l . Single-period subproblem (scenario *k*, period *t*) is given by formulation eq 55. Given the master solution ${\mathrm{q̅}}_{pt}^{l}$ , the recourse LP for each scenario-period pair (*k*, *t*) is

18a $${\mathrm{\phi ̅}}_{kt}^{l}≔\underset{\begin{array}{l} p_{p},b_{j},s_{j} \end{array}}{\min}-\underset{p\in P}{\sum }\sigma_{pt}p_{p}+\underset{j\in J}{\sum }\gamma_{jt}^{k}b_{j}-\underset{j\in J}{\sum }\varphi_{jt}^{k}s_{j}$$

18b $$\begin{array}{ll} \mathrm{s.t.},p_{p}\leq {\mathrm{q̅}}_{pt}^{l} & \forall p \end{array}$$

18c $$\begin{array}{ll} s_{j}\leq A_{jt}^{k},b_{j}\leq D_{jt}^{k} & \forall j \end{array}$$

18d $$\begin{array}{ll} \underset{p}{\sum }\left(\mu_{jp}-\eta_{jp}\right)p_{p}+b_{j}-s_{j}=0 & \forall j \end{array}$$

18e $$\begin{array}{ll} p_{p},b_{j},s_{j}\geq 0 & \forall p,j \end{array}$$

#### Mp Reformulation of the Subproblem

Problem (eq 55) can be written in the compact multiparametric LP form

$$\underset{x}{\min}c^{T}x+\theta^{T}H^{T}x\;\mathrm{s.t.}\;Ax\leq b+F\theta ,x\geq 0$$

by defining

$$x≔\left[\begin{array}{l} p\\ b\\ s \end{array}\right],\theta ≔\left[\begin{array}{l} {\mathrm{q̅}}_{pt}^{l}\\ \gamma_{jt}\\ \sigma_{pt}\\ \varphi_{jt}\\ A_{jt}\\ D_{jt}^{k} \end{array}\right]$$

The mp solver partitions the parameter space Θ={θ|k∈K} into critical regions; inside each region the optimal decision and cost are piecewise affine:

$$x^{*}\left(\theta \right)=A^{v}\theta +b^{v},\;{\mathrm{\phi ̅}}_{kt}\left(\theta \right)=\alpha^{vT}\theta +\beta^{v}$$

### Illustrative Example and Numerical Experiments Setup

The network used to illustrate the stochastic capacity expansion problem is a model adapted from Iyer and Grossmann,^53^ and is shown in Figure 3 which consist of *p* = 3 processes and *J* = 3 chemicals. This case study is well-suited for computational analysis because its structure allows for systematically increasing the problem size by adjusting key parameters. Specifically, we vary the length of the finite planning horizon, *T*, and the number of uncertainty scenarios, *K*. This approach enables a clear evaluation of the computational benefits of our proposed framework as the scale and complexity of the optimization problem grow. For the numerical experiments, we consider four planning horizon lengths, *T* ∈ {5, 10, 25, 50}, and five sizes for the number of scenarios, *K* ∈ {50, 200, 500, 1000, 2000}. While the optimal first-stage decisions may stabilize with fewer scenarios, the larger scenario sets are included specifically to evaluate the computational scalability and performance of the proposed decomposition algorithm under demanding conditions.

**Figure 3:**
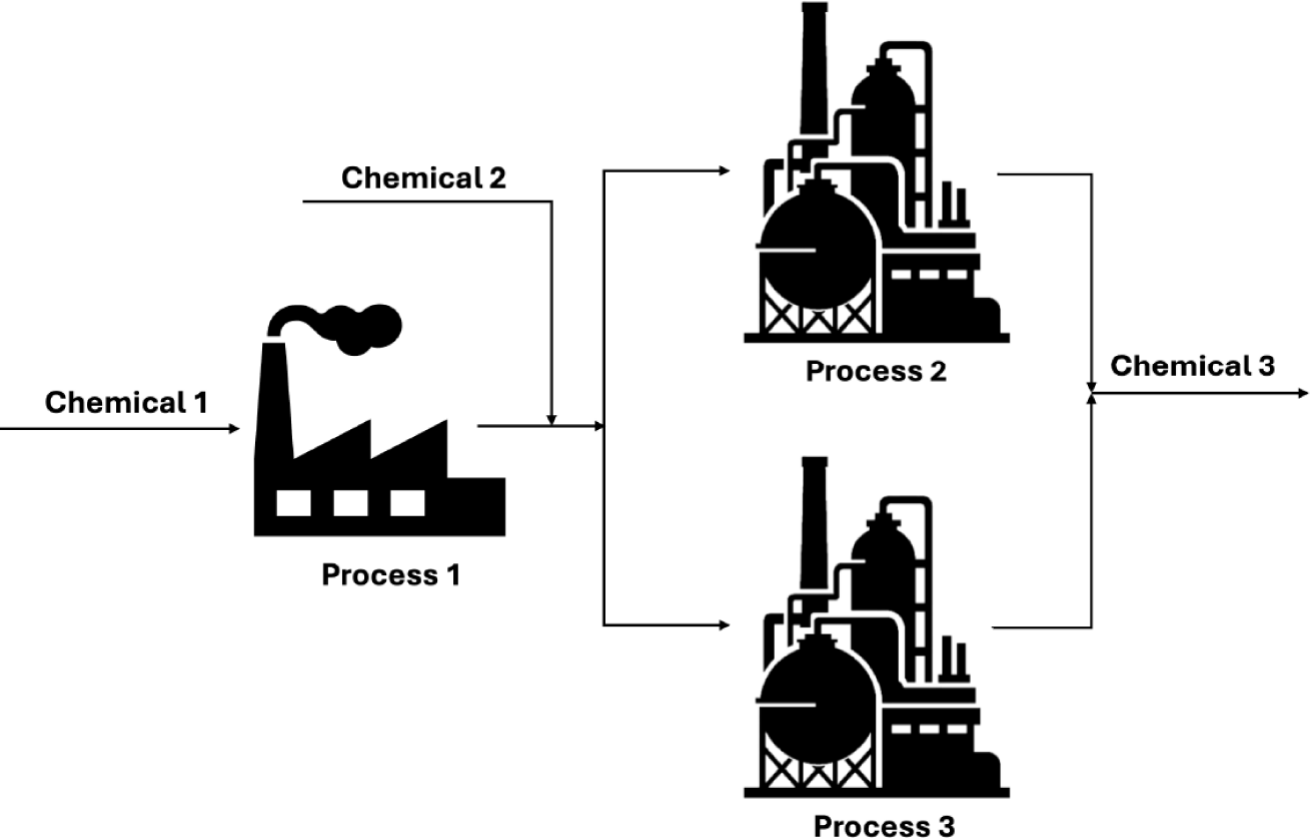
Superstructure of the plant transforming *Chemical 1* and *Chemical 2* into the final product *Chemical 3*. Adapted from Iyer and Grossmann.^53^ Copyright 1998 American Chemical Society.

The stochasticity in the model arises from uncertain market and technological conditions, specifically the demands, availabilities, and prices of the chemicals. Each of these three chemical-related quantities is considered uncertain, yielding a total of six uncertain parameters (i.e., 3 × 2 = 6). In addition, the operating cost parameters σ_*pt*_ for each of the three processes and the first-stage cumulative capacity parameters *q*_*pt*_ are also treated as inputs to the model. This brings the total number of parameters used in the multiparametric (mp) formulation of the subproblem eqs 55 and 35. Table 1 provides a detailed breakdown of the problem size for the case study with a fixed number of scenarios *K* = 2000, processes *p* = 3, and chemicals *J* = 3 across the different time horizons (*T*) tested.

**Table 1:** Computational Statistics for Problem Instances with *K* = 2000 Scenarios

	Number of Time Periods (T)			
Statistic	5	10	25	50
*Monolithic Problem*				
Continuous Variables	90,030	180,060	450,150	900,300
Binary Variables	15	30	75	150
Inequality Constraints	180,075	360,150	900,375	1,800,750
Equality Constraints	30,015	60,030	150,075	300,150
*Single Scenario Subproblem*				
Continuous Variables	45	90	225	450
Inequality Constraints	90	180	450	900
Equality Constraints	15	30	75	150

Although in practice prices, demands, and availabilities are often statistically correlated, we assume for simplicity that they are mutually independent. Each scenario k∈K is treated as an independent realization of uncertainty with a corresponding probability weight π_*k*_, which is used in the expectation operator in the stochastic model.

For completeness, Table 2 lists the mean values employed at period *t* = 4, where the rows correspond to processes *p* = 1, 2, 3 and the columns to time periods *t* = 1, ···, 4. To generate the full scenario set, we sample values from normal distributions centered at these means, using 10% of the mean as the standard deviation for each distribution. The deterministic parameters governing the process are also specified. The lower and upper bounds for capacity expansions are detailed in Table 3, while the stoichiometric consumption and production coefficients for each process are listed in Table 4.

**Table 2:** Mean Parameter Values Used for the Base Case (*p* = 1, 2, 3; *T* = 1,···, 4)

	*t* = 1	*t* = 2	*t* = 3	*t* = 4
α_*pt*_	1.38	1.67	2.22	3.58
	2.72	3.291	4.381	7.055
	1.76	2.13	2.834	4.565
β_*pt*_	85	102.85	136.89	220.46
	73	88.33	117.56	189.34
	110	133.10	177.15	285.31
σ_*pt*_	0.40	0.48	0.64	1.03
	0.60	0.72	0.96	1.55
	0.50	0.60	0.80	1.29
$A_{jt}^{k}$	6.00	7.26	9.66	15.56
	20.00	24.20	32.21	51.87
	0.00	0.00	0.00	0.00
$D_{jt}^{k}$	0.00	0.00	0.00	0.00
	0.00	0.00	0.00	0.00
	30.00	36.30	48.31	77.81
$\gamma_{jt}^{k}$	0.00	0.00	0.00	0.00
	0.00	0.00	0.00	0.00
	26.20	31.70	42.19	67.95
$\varphi_{jt}^{k}$	4.00	4.84	6.44	10.37
	9.60	11.61	15.46	24.90
	0.00	0.00	0.00	0.00

**Table 3:** Lower and Upper Cumulative Capacity Bounds $\left(E_{pt}^{L},E_{pt}^{U}\right)$ and Per-Period Expansion Limit $\left(Q_{pt}^{U}\right)$

	*p* = 1	*p* = 2	*p* = 3
$E_{pt}^{L}$	1	10	10
$E_{pt}^{U}$	6	30	30
$Q_{pt}^{U}$	100	100	100

**Table 4:** Stoichiometric Consumption (*η*_*jp*_) and Production (*μ*_*jp*_) Coefficients

	*p* = 1	*p* = 2	*p* = 3
Consumption coefficients η_*jp*_			
*j* = 1	1.11	0	0
*j* = 2	0	1.22	1.05
*j* = 3	0	0	0
Yield coefficients μ_*jp*_			
*j* = 1	0	0	0
*j* = 2	1	0	0
*j* = 3	0	1	1

### Graph Modeling and Solution

The problem was implemented as a graph model within Plasmo.jl and solved using BD within PlasmoBenders.jl. The resulting OptiGraph is visualized in Figure 4. The first stage problem is placed on a subgraph called $G_{master}$ , which spans all time points (each time point represented as a node). The nodes of this subgraph contain the variables *x*_*pt*_, *y*_*pt*_, and $q_{pt},p\in P,t\in T$ Each of these nodes is also linked to the previous time point ((eq 52)). The time points of each scenario are then added as independent subgraphs, each containing one node. These scenario time points are linked to the master problem by constraints (edges) containing the complicating variable *q*_*pt*_. This results in |K|×|T| subproblem subgraphs that can be solved independently and in parallel.

**Figure 4:**
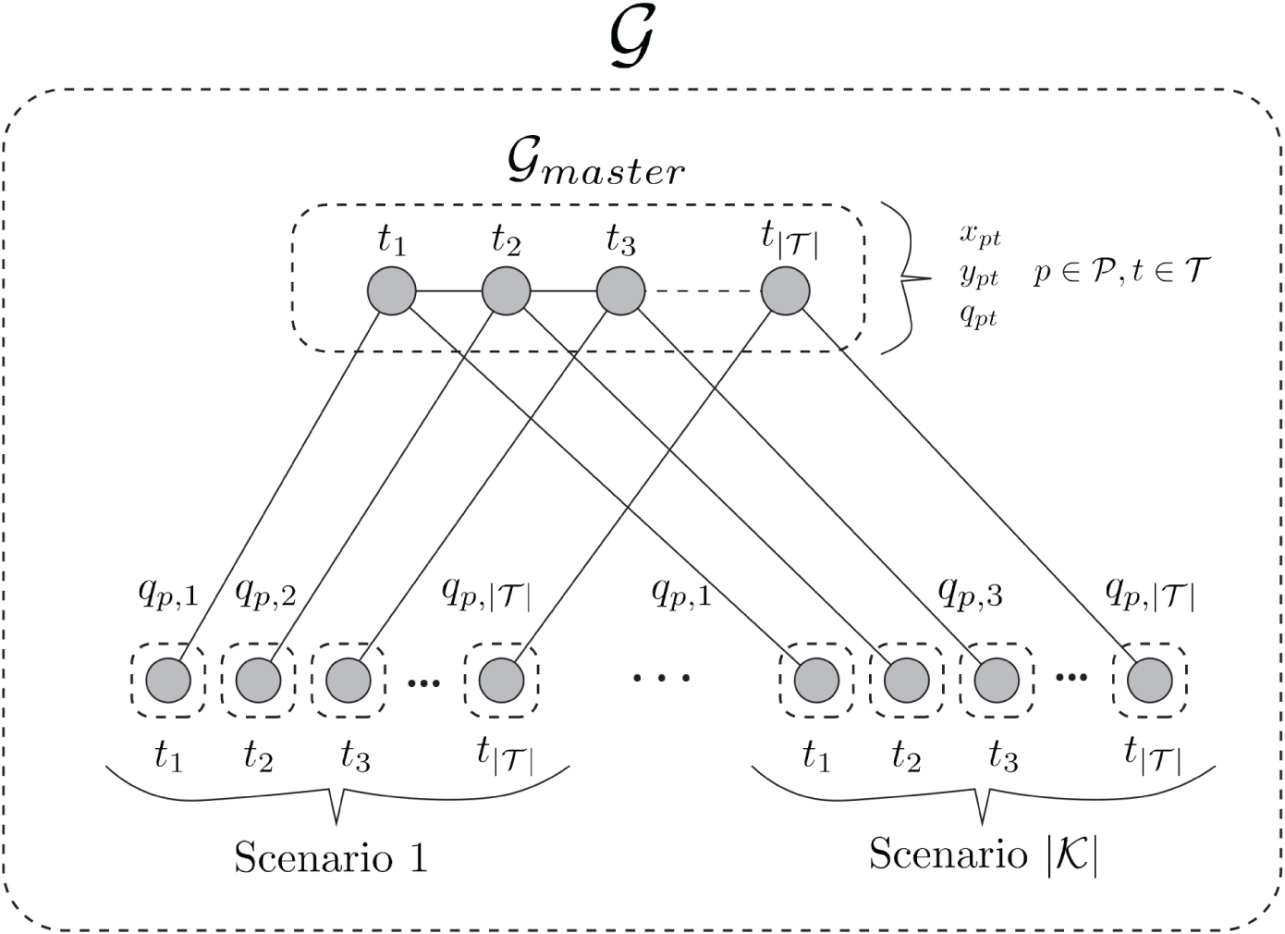
Graph structure of the capacity-expansion planning problem. The master problem subgraph, $G_{master}$ contains the first-stage decisions across all time points. The second-stage capacity decisions, *q*_*pt*_ are linked via linking constraints to the scenario subgraphs at each time point.

The graph model was solved using the mp-data and PlasmoBenders.jl. To do this, the mp problem for this case study was solved once using the PPOPT toolbox.^40^ The graph structure defined above was passed to PlasmoBenders.jl, which automatically detects the subproblems once the master problem is set. A classic MILP solver (HiGHS or Gurobi) was set on the master problem subgraph, and the custom solver was set on each of the subproblem subgraphs. The resulting mp data was set within the custom solver used on each subproblem subgraph, and Benders was applied to solve each problem.

To analyze the behavior of mp-Benders, we tested the framework using both HiGHS^54^ and Gurobi^55^ as the MILP solver used for solving the master problem and the custom mp solver on the subproblems. We also solved the subproblems using HiGHS and Gurobi for comparison with mp. Gurobi is a state-of-the-art commercial solver, but it requires a proprietary license. To ensure our results are reproducible for researchers who may not have access to commercial software, we also included HiGHS, a leading open-source solver. All tests were run using PlasmoBenders.jl for consistency. Solves were done with both multicut and single-cut Benders approaches.

## Results and Discussion

### Computational Results: Multicut vs Single-Cut Benders Decomposition

Figure 5a–d illustrate the computational results for the multicut Benders decomposition with planning horizons *T* = 5, 10, 25, 50. The subproblem solution time is reported for varying numbers of scenarios *K* = 50, 200, 500, 1000, 2000, comparing the mp-based critical region querying method (“mp-Benders”) to direct solves using Gurobi (“Gurobi-Benders”) and HiGHS (“HiGHS-Benders”). Across all cases, the mp-Benders approach consistently yields faster subproblem solution times, particularly as the number of scenarios increases. These performance improvements are primarily due to the avoidance of repeated LP solves by querying precomputed affine solutions from critical regions. It is important to note that the reported subproblem solution times for the mp-Benders case include the one-time overhead of computing the critical regions (offline). As a result, for smaller scenario sizes, the mp-Benders approach may not outperform the direct Gurobi or HiGHS subproblem solves, since the relative impact of the critical region generation time is more significant in such cases.

**Figure 5:**
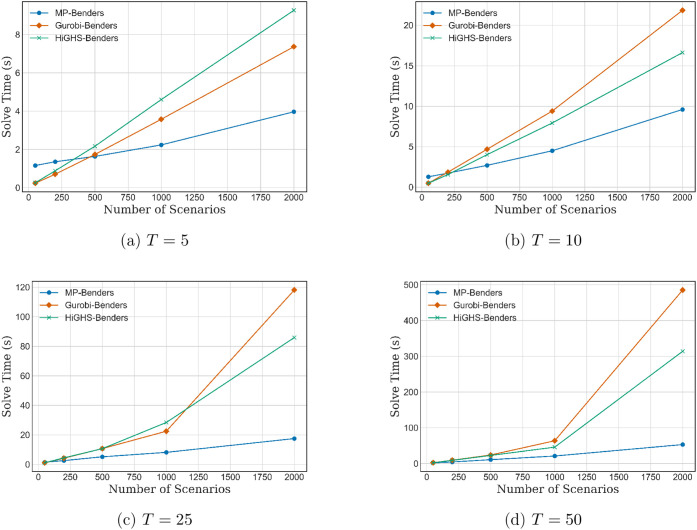
Subproblem solve time vs number of scenarios for different time horizons *T* (multicut).

Figure 6a–d presents the results for the single-cut Benders decomposition variant. The primary motivation for examining this variant is to assess the performance advantages of mp-Benders surrogates in settings that require a large number of Benders iterations, as is typical with single-cut formulations. Notably, the subproblem solve time using mp-Benders is significantly lower than that of Gurobi- or HiGHS solvers, and this difference is pronounced compared to the multicut case. This observation suggests that the computational benefits of mp surrogates become increasingly prominent in scenarios where the decomposition algorithm must generate a greater number of cuts to converge.

**Figure 6:**
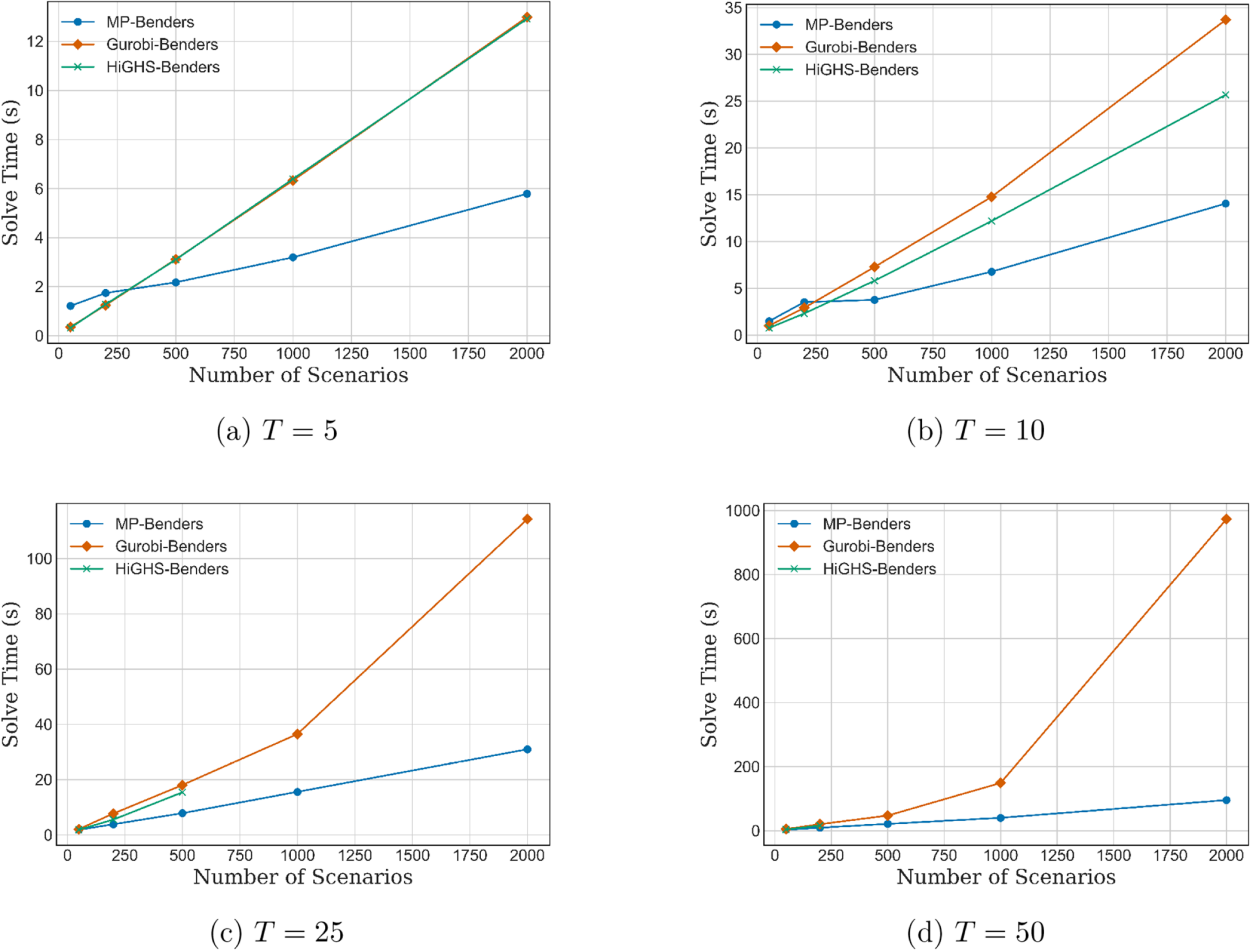
Subproblem solve time vs number of scenarios for different time horizons *T* (single-cut).

Nevertheless, for this particular case study, all single-cut variants- mp-Benders, Gurobi-Benders, and HiGHS-Benders exhibit longer overall solution times relative to their multicut counterparts. This outcome is primarily attributed to the slower convergence of the single-cut scheme, which aggregates recourse information and thus provides less informative feedback per iteration.

It is also important to highlight that for the large planning horizons (*T* = 25 and *T* = 50), the HiGHS-Benders implementation failed to solve problem instances with high scenario counts (*K* ∈ {500, 1000, 2000}). Despite multiple attempts, we were unable to isolate the root cause of these failures, which we suspect stem from numerical instability or solver tolerance thresholds. Consequently, the affected data points are omitted from the corresponding plots [Fig fig6]c,d.

### Interpretability of Benders Decomposition Using Mp Programming

The proposed mp framework provides a direct and analytical way to achieve interpretability. The mp formulation provides an explicit, geometric partitioning of the parameter space into critical regions, where each region is associated with a unique affine decision and value function. These critical regions can be viewed as an analytical form of the recourse-based scenario clustering proposed by Rathi et al.;^32^ all scenarios (parameter vectors) that fall within a single critical region are, by definition, a “cluster” that shares the same set of active constraints and is governed by the same affine recourse function. This allows us to understand entire classes of scenarios at once. Furthermore, by inspecting the affine solution function (x* = *A* ^*v*^θ + *b* ^*v*^) within each region, we can perform an analysis analogous to recourse reduction. The components of the matrix *A* ^*v*^ reveal which recourse variables are sensitive to changes in first-stage decisions and uncertain parameters, thereby identifying the most “principal” or flexible decisions for that entire region.

By embedding this structure into the Benders framework, we gain the ability to visualize how the master problem iterates navigate this explicit critical region landscape. To illustrate this, we analyze the Benders solution process for a representative problem instance with a time horizon of *T* = 5 and a set of *K* = 500 scenarios. Figures 7–10 depict the trajectory of the first-stage capacity decisions (*q*_*pt*_) as they evolve over the Benders iterations. Each point represents a solution from the master problem, which is guided by cuts from all 500 scenarios. Each subplot corresponds to the decision variable for a specific time period (*t* ∈ {1, ··· , 5}), showing how the algorithm searches the critical region space for that particular decision. This visualization reveals several key insights:

- **Convergence behavior:** In the multicut case, the iterates tend to converge more directly toward the final solution, traversing fewer critical regions. This aligns with the theoretical observation that multicut Benders converges in fewer iterations due to richer feedback from the subproblems.
- **Decision consistency:** Within certain time steps (e.g., *t* = 3 or *t* = 4), the iterates remain confined to a single or a small number of regions. This suggests robustness of the recourse structure with respect to small perturbations in the master decision, i.e., the recourse decisions exhibit local linear stability.
- **Aggregation effect:** The single-cut formulation requires more iterations and exhibits more oscillatory behavior, with the decision path traversing a greater number of distinct regions. This reflects the loss of resolution caused by aggregating all subproblem feedback into a single surrogate.
- **Policy transparency:** Because each region corresponds to a fixed active set of constraints, we can determine *why* a particular recourse policy (the affine function) is optimal for an entire class of scenarios (the critical region) by identifying the binding operational constraints. This allows for a richer postoptimal analysis, identifying which constraints dominate solution behavior under different uncertainty realizations.

**Figure 7:**
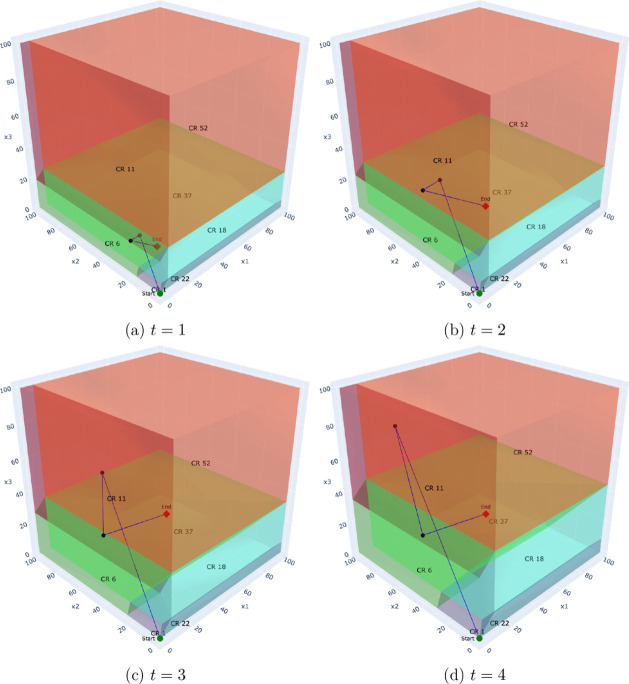
Multicut: Evolution of first-stage decisions (*q*_*pt*_) over Benders iterations for a problem with *T* = 5 and *K* = 500. Each subplot shows the iteration trajectory for the decision in a specific time period (*t* = 1 to *t* = 4).

**8:**
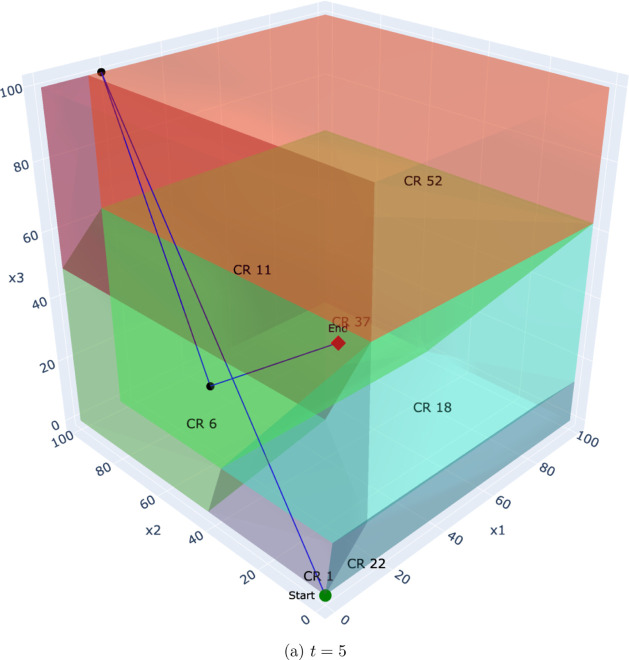
Multicut: Evolution of the first-stage decision (*q*_*pt*_) over Benders iterations for the final time period, *t* = 5.

**9:**
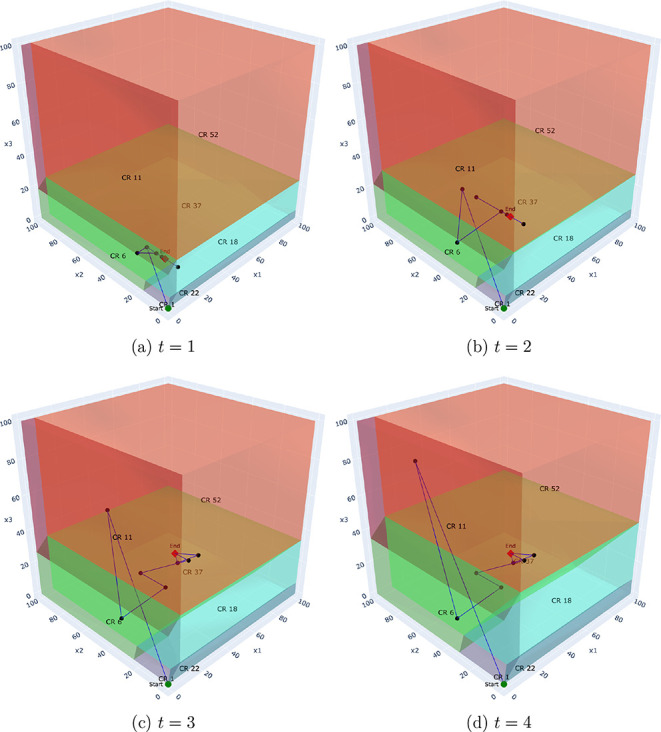
Single-cut: Evolution of first-stage decisions (*q*_*pt*_) over Benders iterations for a problem with *T* = 5 and *K* = 500. Each subplot shows the iteration trajectory for the decision in a specific time period (*t* = 1 to *t* = 4).

**Figure 10:**
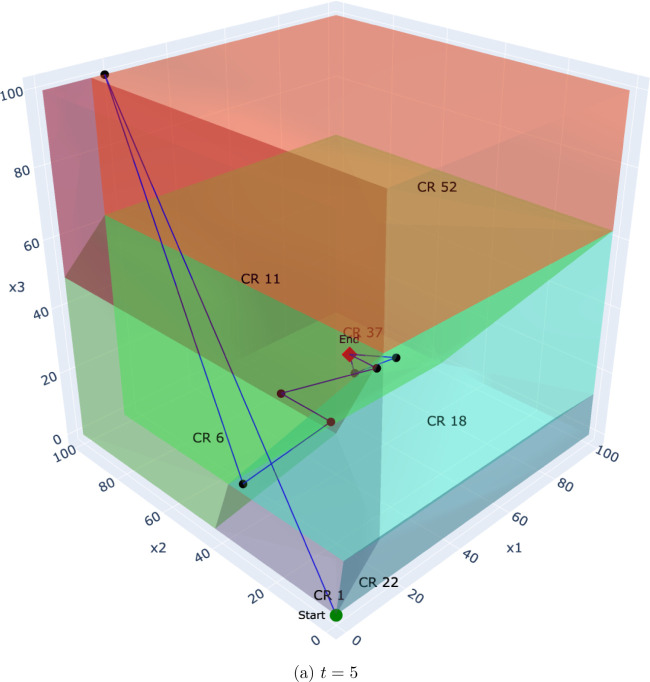
Single-cut: Evolution of the first-stage decision (*q*_*pt*_) over Benders iterations for the final time period, *t* = 5.

Taken together, these insights offer a new perspective on stochastic optimization algorithms: not just as black-box iterative solvers, but as structured geometric processes navigating a known (although high-dimensional) partition of the parameter space. This geometric lens can support better debugging, policy interpretation, and even sensitivity analysis with respect to scenario inputs or problem data, directly addressing the need for greater explainability in stochastic optimization. While the computational speedups are most pronounced for a large number of scenarios, the framework remains competitive for medium-sized problems; moreover, the enhanced solution interpretability offered by critical region tracking provides a significant advantage that is independent of problem scale.

Figure 11 shows the evolution of the upper and lower bound for both multicut and single-cut cases for a representative problem instance. This result confirms that the mp-based Benders approach converges to the optimal solution. This also illustrates that mp surrogates provide optimality guarantees, since primal and dual variables are optimal (not approximate).

**Figure 11:**
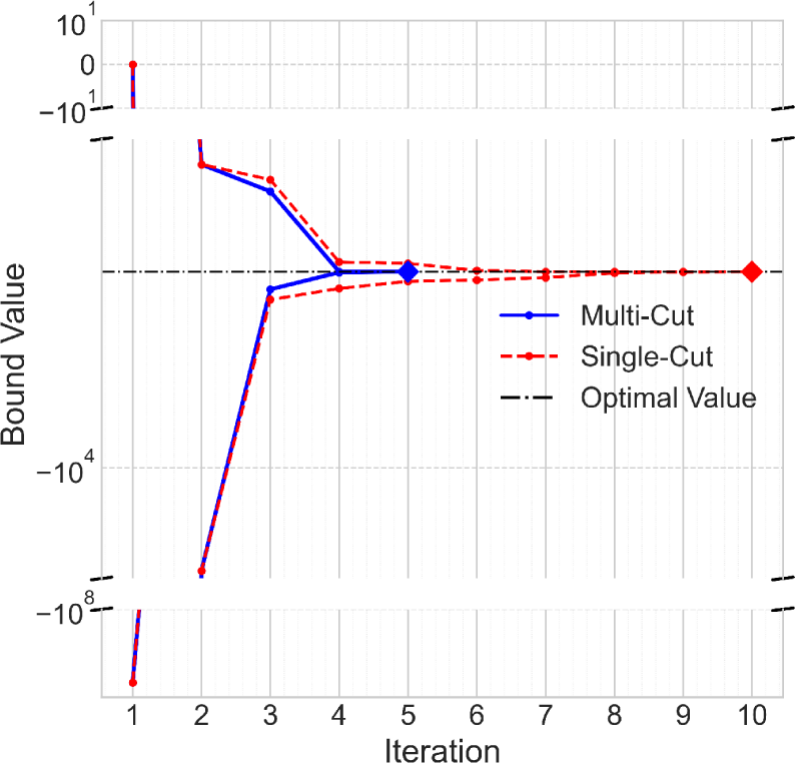
Bound convergence for multicut (blue) and single-cut (red) Benders algorithms with *T* = 5 and *K* = 500. Diamond markers indicate convergence to the optimal value (black dash-dot line). Note: The results shown are same for the mp-Benders method using Gurobi.

### Benefits of Graph-Based Software Framework

The graph modeling approach provides a flexible and modular framework for constructing and visualizing complex optimization problems. Plasmo.jl allows for treating pieces of the optimization problem (subgraphs) as independent optimization problems, making it simpler to define decomposition algorithms that solve subproblems of a larger, overall problem. Furthermore, this allows for performing custom tasks on a single subgraph of the optimization problem without altering the overall problem structure or changing other subgraphs in the optimization problem. For instance, this could facilitate replacing a subgraph with a surrogate model or, as we did in this work, setting a custom solver on individual subgraphs that acted as an mp surrogate model.

## Conclusions and Future Work

We presented a computational framework for Benders decomposition that embeds multiparametric programming (mp) surrogates within a graph representation of structured optimization problems. By integrating mp-LP surrogates of the graph node subproblems, we replace expensive repeated LP solves with region look-ups and affine evaluations, leading to substantial runtime reductions. Our implementation in PlasmoBenders.jl demonstrates the flexibility of graph decomposition and the interpretability benefits offered by critical region tracking. Computational experiments on a stochastic capacity expansion case study confirmed that the proposed mp-Benders approach provides consistent speedups across varying time horizons and scenario sizes, especially in the multicut variant.

We acknowledge key limitations of the mp-based surrogate approach related to its scalability. As the number of uncertain parameters in a subproblem grows, the number of critical regions can increase in a combinatory manner, making the generation of the mp solution computationally expensive. Furthermore, the total computational effort also scales with the number of structurally distinct subproblems; the case study presented here benefited significantly from subproblem homogeneity, whereas applications with many heterogeneous subproblems would require a correspondingly larger mp solution effort. These offline costs create a practical trade-off, and a valuable direction for future work is to perform a detailed scalability analysis to identify the threshold where this offline effort outweighs the online speedups gained in the Benders decomposition. For the case study under consideration in this work, altering the number of parameters to perform such an analysis would require redesigning the process itself, so we leave this broader investigation for future studies. To address these scalability challenges directly, we aim to develop more scalable, modular mp surrogates, where each large subproblem may be approximated by multiple smaller mp models. This could mitigate the explosion in region count by partitioning the parameter space adaptively.

Furthermore, our current framework is centered around explicit mpLP surrogates. A particularly important direction will be the extension of our framework to multiparametric mixed-integer linear programs (mp-MILPs), which would enable the exact treatment of subproblems with integer recourse variables. We also intend to extend the surrogate capabilities of the Plasmo.jl and PlasmoBenders ecosystem by incorporating alternative surrogate models from the machine learning domain such as decision trees, input convex neural networks (ICNNs), and other neural architectures as plug-and-play surrogates within the graph-based modeling approach. This will enable hybrid surrogate strategies that trade off interpretability, scalability, and approximation quality depending on the application context. The current work employs a multitree approach; a key future development will be to implement a single-tree Benders algorithm that adds cuts as lazy constraints within a single branch-and-cut tree, which can be more efficient for MILP master problems in PlasmoBenders. By building toward a unified, modular, and extensible surrogate-enhanced optimization framework, we hope to broaden the applicability of decomposition methods to real-world large-scale optimization problems.

## Nomenclature

Symbol: Description
G: OptiGraph abstraction
N(G): Set of nodes in graph G
E(G): Set of edges in graph G
*x*_*n*_: Decision variables on node *n*
$X_{n}$: Feasible set for decision variables on node *n*
*f*(·): Objective function
*g*(·): Constraint function
*x*_*m*_: Master problem decision variables
*x*_*m*_: Subproblem variables for subproblem *w*
*W*: Set of subproblems
$c_{m},c_{w}$: Cost vectors for master and subproblems
$C_{w},D_{w},q_{w}$: Constraint matrices and vectors
α_*w*_: Cost-to-go variables
${\underset{―}{\phi }}_{m}^{i}$: Master problem objective value at iteration *i*
${\overset{―}{\phi }}_{w}^{i}$: Subproblem objective value for subproblem *w* at iteration *i*
${\overset{―}{x}}_{m}^{i}$: Master problem solution at iteration *i*
$\lambda_{w}^{i}$: Dual variables for subproblem *w* at iteration *i*
*UB* ^*i*^: Upper bound at iteration *i*
*LB* ^*i*^: Lower bound at iteration *i*
θ: Vector of uncertain/unknown parameters
*x*: Vector of decision variables in mp problem
*J* * (*θ*): Optimal objective value as a function of θ
Θ: Compact polytope of bounded uncertain parameters
*z*(θ): arametric objective function in mp-LP
*c*, *H*: Matrices and vectors in mp-LP objective function formulation
*A*, *b*, *F*: Matrices and vectors inequality constrains in mp-LP formulation
*A*_*eq*_, *b*_*eq*_, *F*_*eq*_: Matrices and vectors for equality constraints in mp-LP
*A*_θ_, *b*_θ_: Matrix and vector defining the parameter bounds space Θ
*v*: ndex for a critical region
*n*_*CR*_: Total number of critical regions
*x*_*_: ptimal solution of the mp problem
*A* ^*v*^, *b* ^*v*^: Affine function matrices for solution in critical region *v*
*E* ^*v*^, *f* ^*v*^: matrices defining critical region *v*
P: Set of processes
J: Set of chemicals
T: Set of planning periods
K: Set of uncertainty scenarios
*x*_*pt*_: Capacity expansion decisions for process *p* in period *t*
*y*_*pt*_: Binary decision to expand process *p* in period *t*
*q*_*pt*_: Cumulative capacity expansion for process *p* in period *t*
*p*_*pt*_: Production level of process *p* in period *t*
*b*_*jt*_: Amount of chemical *j* purchased in period *t*
*s*_*jt*_: Amount of chemical *j* sold in period *t*
α_*pt*_, β_*pt*_: Variable and fixed cost coefficients for expansion
σ_*pt*_: Variable operating cost for process *p* in period *t*
$A_{jt}^{k}$: Availability of chemical *j* in period *t* under scenario *k*
$D_{jt}^{k}$: Demand of chemical *j* in period *t* under scenario *k*
$\gamma_{jt}^{k}$: Purchase price of chemical *j* in period *t* under scenario *k*
$\varphi_{jt}^{k}$: Selling price of chemical *j* in period *t* under scenario *k*
η_*jp*_: Stoichiometric consumption coefficient of chemical *j* in process *p*
μ_*jp*_: Stoichiometric yield coefficient of chemical *j* in process *p*
$E_{pt}^{L},E_{pt}^{U}$: Lower and upper cumulative capacity bounds
$Q_{pt}^{U}$: Per-period expansion limits
π_*k*_: Probability of scenario *k*
*V*(*q*,ξ): Recourse value function for a given plan *q* and realization ξ
ξ: Realization of uncertain parameters $\left(A_{jt}^{k},D_{jt}^{k},\gamma_{jt}^{k},\varphi_{jt}^{k}\right)$
